# Comparison of spatial working memory in children with prenatal alcohol exposure and those diagnosed with ADHD; A functional magnetic resonance imaging study

**DOI:** 10.1186/1866-1955-4-12

**Published:** 2012-05-18

**Authors:** Krisztina L Malisza, Joan L Buss, R Bruce Bolster, Patricia Dreessen de Gervai, Lindsay Woods-Frohlich, Randy Summers, Christine A Clancy, Albert E Chudley, Sally Longstaffe

**Affiliations:** 1 Department of Physiology, University of Manitoba, 432 Basic Medical Sciences Bldg, 745 Bannatyne Ave, Winnipeg, MB R3E 0J9, Canada; 2 Department of Psychology, University of Manitoba, P404 Duff Roblin Bldg, 190 Dysart Rd, Winnipeg, MB R3T 2N2, Canada; 3 National Research Council of Canada, Institute for Biodiagnostics, 435 Ellice Avenue, Winnipeg, MB R3B 1Y6, Canada; 4 Department of Psychology, University of Winnipeg, Winnipeg, Canada; 5 Division of Rehabilitation Psychology, Seattle Children’s Hospital, Seattle, WA 98105, USA; 6 Department of Biochemistry and Medical Genetics, University of Manitoba, 336 Basic Medical Sciences Bldg, 745 Bannatyne Ave, Winnipeg, MB R3E OJ9, Canada; 7 Department of Pediatrics and Child Health, CE-203 Children’s Hospital, Health Sciences Centre, 840 Sherbrook St, Winnipeg, MB R3A 1S1, Canada

**Keywords:** Fetal Alcohol Spectrum Disorder (FASD), Alcohol related neurodevelopmental disorder (ARND), Attention-deficit/hyperactivity disorder (ADHD), Functional magnetic resonance imaging (fMRI), Spatial working memory, White matter, Gray matter, Diffusion tensor imaging (DTI), Region of interest (ROI)

## Abstract

**Background:**

Alcohol related neurodevelopmental disorder (ARND) falls under the umbrella of fetal alcohol spectrum disorder (FASD), but individuals do not demonstrate the facial characteristics associated with fetal alcohol syndrome (FAS), making diagnosis difficult. While attentional problems in ARND are similar to those found in attention-deficit/hyperactivity disorder (ADHD), the underlying impairment in attention pathways may be different.

**Methods:**

Functional magnetic resonance imaging (fMRI) of a working memory (1-back) task of 63 children, 10 to 14 years old, diagnosed with ARND and ADHD, as well as typically developing (TD) controls, was conducted at 3 T. Diffusion tensor imaging (DTI) data were also acquired.

**Results:**

Activations were observed in posterior parietal and occipital regions in the TD group and in dorsolateral prefrontal and posterior parietal regions in the ARND group, whereas the ADHD group activated only dorsolateral prefrontal regions, during the working memory component of the task (1-back minus 0-back contrast). The increases in frontal and parietal activity were significantly greater in the ARND group compared to the other groups. This increased activity was associated with reduced accuracy and increased response time variability, suggesting that ARND subjects exert greater effort to manage short-term memory load. Significantly greater intra-subject variability, demonstrated by fMRI region-of-interest analysis, in the ADHD and ARND groups compared to the TD group suggests that moment-to-moment lapses in attention contributed to their poorer task performance. Differences in functional activity in ARND subjects with and without a diagnosis of ADHD resulted primarily from reduced activation by the ARND/ADHD + group during the 0-back task. In contrast, children with ADHD alone clearly showed reduced activations during the 1-back task. DTI analysis revealed that the TD group had significantly higher total tract volume and number of fibers than the ARND group. These measures were negatively correlated with errors on the 1-back task, suggesting a link between white matter integrity and task performance.

**Conclusions:**

fMRI activations suggest that the similar behavior of children with ARND and ADHD on a spatial working memory task is the result of different cognitive events. The nature of ADHD in children with ARND appears to differ from that of children with ADHD alone.

## Background

Fetal alcohol spectrum disorder (FASD), the umbrella term describing the spectrum of ethanol teratogenesis in humans, is a common cause of developmental disability [1]. At one end of this spectrum is the subset of individuals with fetal alcohol syndrome (FAS), characterized by growth deficiency, facial abnormalities, and central nervous system (CNS) damage. At the other end of the spectrum are individuals with behavioral and cognitive deficits termed alcohol related neurodevelopmental disorder (ARND), a diagnosis which is not associated with physical stigmata. While outwardly visible characteristics such as growth deficiency and facial phenotype are easily defined, CNS damage manifested as anatomical, cognitive and behavioral deficits is diverse [2-4]. IQ is often low in children with obvious anatomical changes, whereas children with prenatal alcohol exposure and average IQ tend to have no detectable anatomical abnormalities [5].

Without an appropriate diagnosis, children with FASD may be denied access to the treatment and services they require. Diagnosis of an FASD is a challenge, especially in adults and children with ARND, as many of the symptoms are non-specific and no consistent neurodevelopmental profile has been established. Individuals with ARND do not demonstrate the facial features characteristic of fetal alcohol syndrome (FAS), but still have neurodevelopmental, cognitive or behavioral abnormalities [1,6] that overlap with other conditions, such as Attention Deficit/Hyperactivity Disorder (ADHD) [7-9]. Attentional problems found in FASD are generally similar to those in individuals diagnosed solely with ADHD [10,11]. Confounding the diagnostic challenge further is the likelihood of the existence of comorbid conditions in some individuals [7,9]. Because of the behavioral similarities between children with ARND and ADHD, differential diagnosis based on task performance and behavioral assessment may not be sufficient to distinguish between these groups of children.

While the symptoms of ARND and ADHD are similar, the underlying impairments in cognitive pathways may be different, as they have independent origins. Furthermore, ADHD among children with FASD appears to differ somewhat from that in the general population, having earlier onset and greater prevalence of the inattentive subtype [7]. In one study of attention, children with ADHD had the most difficulty focusing and sustaining attention, whereas children with FAS performed most poorly on the encoding of new information [12]. A study of verbal learning and memory also concluded that children with FASD had difficulty encoding new information, whereas children with ADHD had a deficit in retrieving the information once it was learned [13].

Working memory, the short-term maintenance of information in an active state for updating or manipulation [14], is one component of executive function and underlies successful reasoning and problem solving. Working memory deficits have been documented in subjects with FASD [15-18] and have been well established in the ADHD population [19,20]. Two functional magnetic resonance imaging (fMRI) studies of children with FASD [21,22] have reported increased activation, relative to control children, across the network of brain regions expected for a working memory task, while a third study [23] found increased activation in inferior and middle frontal cortices and decreased activation in superior frontal and parietal regions. Meta-analysis of the neural correlates of a variety of tasks in subjects with ADHD indicate that hypofrontality, that is weak activation across the frontal cortex and cingulate, is the most consistent finding [24]. Divergent fMRI findings despite similar behavior deficits suggest that the mechanisms of the impairment differ, and that fMRI may offer insight into the nature of the working memory deficits in these two groups.

Within the framework of a larger study investigating differential executive function in children with ARND and ADHD, brain activations during a spatial working memory task were assessed by fMRI. It is hypothesized that brain activation patterns of children with ARND and ADHD will differ from those of a control population, and from each other, despite the possibility of negligible differences in task performance.

## Methods

### Recruitment

The experimental protocol was reviewed and approved by the affiliated ethics boards, including the National Research Council Ottawa Research Ethics Board (REB), the University of Manitoba Human REB, and the University of Winnipeg REB. Written informed consent was obtained by parents or legal guardians and all subjects also provided assent to participate in the study. Subjects 10 to 14 years old with ARND (n = 23) were recruited through the Clinic for Alcohol and Drug Exposed Children (CADEC) using very conservative diagnostic criteria [1]. Children with ADHD (n = 20) were referred through pediatric physician offices who are known to specialize in ADHD and also by Dr. Longstaffe, a member of our multidisciplinary team. Typically developing children (TD, n = 21) were recruited through poster advertisements in local community centers and pediatrician offices, and matched with ADHD and ARND subjects on the basis of gender and age. Demographics for the subjects are presented in Table 1. Medical documentation from the parents and/or guardians was used to confirm the diagnoses of all participants.

**Table 1:** Subject characteristics

**Measure**	**TD (n = 21)**		**ARND (n = 22)**		**ADHD (n = 20)**		**Significance**^**a**^	***Post-hoc*tests**
	Mean	Std Dev	Mean	Std Dev	Mean	Std Dev		
Age (years)	12.60	1.29	12.21	1.63	11.99	1.32	F = 0.950 *P* = 0.392	
Gender (% male)	76.2		63.6		90		χ^2^ = 4.014 *P* = 0.134	
Household Income ($)^b^	70,842	15,302	52,989	19,690	66,167	27,416	F = 3.965 *P* = 0.024	TD > ARND^c^*P* = 0.006
Full Scale IQ	107.81	13.08	74.38	11.69	96.55	16.87	F = 30.968 P <0.001	TD > ARND^d^*P* <0.001
								ADHD > ARND *P* <0.001

^a^*P* value cutoff = 0.05; ^b^Average income in the postal code of residence; ^c^Games-Howell test; ^d^Tukey’s HSD test. ADHD, attention deficit/hyperactivity disorder; ARND, alcohol related neurodevelopmental disorder; IQ, intelligence quotient: Std Dev, standard deviation; TD, typically developing.

TD subjects were excluded from the study if they had been referred to a psychiatrist for behavioral problems. TD and ADHD subjects were excluded if their mothers had consumed alcohol or drugs during pregnancy. All children were screened prior to participation in the study using a standard MRI questionnaire. Subjects in all groups were excluded for contraindications to MRI scanning (braces, metal implants, and so on). The Wechsler Intelligence Scale for Children – Fourth Edition (WISC-IV) was not administered to any child who had performed the WISC within the past two years. Prior to imaging, children were assessed in a mock scanner to ensure they could lie sufficiently still to obtain good MR imaging. If it was deemed that they were unable to lie sufficiently still for at least 10 minutes, they were excluded from the study.

### Medication

All children were free of stimulant medication (methylphenidate, dextroamphetamine, and caffeine) for at least 36 hours prior to the fMRI acquisition and for the psychological assessments. Children who were taking Strattera, Risperidone, or other non-stimulant medication stayed on these medications for the study.

No subjects in the TD group were taking either stimulant or non-stimulant medications. In the ADHD group, five subjects were unmedicated, eleven were taking stimulants, one was taking non-stimulants (strattera and risperidone), and three were taking both stimulants and risperidone. In the ARND group, four subjects were unmedicated, eight were taking stimulants, two were taking non-stimulants (atomoxetine, quetiapine), and eight were taking stimulants and risperidone, three of whom were also taking fluxotine, olanzapine, or quetiapine.

Among the ARND subjects diagnosed with ADHD (ARND/ADHD+), one was unmedicated, six were taking stimulants, and four were taking stimulants and risperidone, one of whom was also taking olanzapine. Among the ARND subjects without ADHD (ARND/ADHD-), three were unmedicated, three were taking stimulants, two were taking non-stimulants (quetiapine, atomoxetine), and three were taking stimulants and risperidone.

### Comorbidities

FASD is associated with many comorbid disorders [9]. The ARND group included one child with learning disability, conduct disorder and global delay, one child with oppositional defiant disorder (ODD), one child with depression, and one child with attachment disorder. ADHD is a common comorbid disorder with FASD [7,8] and eleven subjects in the ARND group, including the four subjects with the disorders listed above, had been diagnosed with this disorder. Among children with FASD, ADHD is more likely to be the earlier-onset, inattention subtype, with comorbid developmental psychiatric and medical conditions such as anxiety, mood, conduct or explosive disorders [7]. There is controversy over whether ADHD is, in fact, comorbid with ARND, since attention is one of the relevant brain domains in the diagnosis of ARND [1]. Since these disorders are frequently present in FASD-affected individuals, it is not possible to exclude participants with them without biasing the sample. Of the 22 subjects in the ARND group, 11 had no documented comorbid disorders.

ADHD is associated with many learning and behavioral disorders [7]. The ADHD group included four subjects with learning disabilities (math and/or reading), and one subject with ODD. None of the subjects in the TD group were diagnosed with any learning or behavioral disorders.

### Psychological assessment

A battery of standardized psychological tests was administered to all subjects. These were selected to correspond to measures from a series of fMRI tasks (including working memory, attention and response inhibition), conducted as part of a larger study, and included the WISC-IV and the Conners’ Continuous Performance Test II (CPT-II). Teachers and parents or caregivers completed several behavior rating forms, including the Conners Rating Scales, the Child Symptom Inventory – Fourth Edition (CSI-4) or Adolescent Symptom Inventory – Fourth Edition (ASI-4) depending on the age of the child, and the Behavior Rating Inventory of Executive Function (BRIEF). Only components related to working memory are described here.

### fMRI tasks

A series of fMRI tasks were administered over a period of two days, including tasks of working memory, attention and response inhibition. All components of related tasks were administered on the same day. Only components of the working memory task and behavioral measures related to working memory are presented here; results from the other tasks and corresponding behavioral measures are forthcoming.

**Figure 1:**
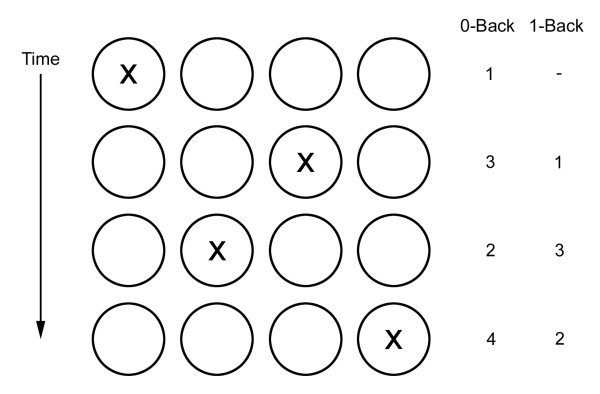
**Task design.** Each row of circles corresponds to the display for a single trial, which appears following a fixation cross. Correct responses for the 0-back and 1-back tasks are indicated to the right.

Within a larger battery of behavioral tests, two event-related fMRI n-back tasks were completed by each subject to assess working memory. The 0-back (control) task consisted of the presentation of four circles in a horizontal array as shown in Figure 1, one of which contained an ‘X.’ Subjects indicated the location of the ‘X’ by pressing a button with the right hand corresponding to its location. A total of 48 stimuli were presented in pseudorandom order and subjects were instructed to respond as quickly and accurately as possible to all trials. A fixation signal was presented immediately before the stimuli. Stimuli were presented for 500 ms, followed by a jittered interstimulus interval (ISI) of 4 to 14 seconds during which responses were recorded. Response times were calculated from the start of the ISI. The 1-back task was similarly structured, consisting of 64 trials, except that subjects were instructed to respond by indicating the location of the stimulus on the previous trial. Reaction times and accuracy were recorded for both tasks. Unlike conventional n-back tasks in which the subject is asked to respond to occasional appearance of target stimuli, this task was structured so that responses were required on every trial to minimize lapses in attention. Children were trained on all fMRI tasks prior to entering the MRI. Children who were unable to perform the tasks sufficiently well were excluded from that fMRI portion of the study.

### Statistics

Statistics were calculated using Statistical Package for Social Sciences (SPSS 17.0, IBM, Armonk, NY USA). In many cases, task performance data could not be transformed to meet the assumptions of parametric statistics. Instead of repeated-measures analysis of variance (ANOVA), one-way ANOVAs followed by Games-Howell *post-hoc* tests were calculated, as well as paired *t*-tests as necessary. Instead of ANCOVAs, Z scores were calculated. In all cases, *P* values were corrected for multiple comparisons.

### Magnetic resonance imaging

All imaging experiments were conducted on a 3 T Siemens TIM TRIO MRI system (Siemens, Erlangen, Germany). Functional MRI data were acquired using a single shot, gradient echo, echo planar imaging sequence with a matrix size of 64 x 64, field of view of 24 cm, echo time of 30 ms, repetition time of two seconds, 28 contiguous 5 mm thick slices oriented parallel to the anterior commissure – posterior commissure (AC-PC) line, resulting in full brain coverage and a voxel size of 3.75 X 3.75 X 5 mm. A total of 170 and 225 volumes were acquired for the 0-back and 1-back tasks, respectively. Standard T1-weighted images were acquired with the same slices selected as for the fMRI experiments.

Functional MRI data were analyzed using SPM5 (http://www.fil.ion.ucl.ac.uk/spm/). Data were converted to SPM analyze format, reoriented, realigned, normalized to the Montreal Neurological Institute (MNI) echo-planar imaging (EPI) template, and smoothed with a 6 mm isotropic kernel. Activation was assessed through the change in signal intensity and volume of activated clusters following the modeled time course over the whole brain. Data were analyzed using fMRI scans corresponding to trials in which the subjects responded correctly. For three subjects in the ADHD group and two subjects in the ARND group, scans corresponding to times when subjects were not engaged in the task (on average, for these five subjects, 18 % of the data for the 1-back task) were manually removed from the analysis. One subject diagnosed with ARND was excluded for performance below the cutoff of 32 correct responses on the 1-back task, resulting in an *n* of 22 subjects for this group. Within-group analysis was conducted using one-sample *t*-tests using the individual subjects’ images for the contrasts of interest. Between-group comparisons were completed using ANOVAs (*P* = 0.05) to determine whether group differences existed, and two-sample, one-tailed *t*-tests (*P* = 0.0167, corrected for multiple comparisons) to compare the groups pairwise. Significance levels were corrected for multiple comparisons using cluster correction, yielding family-wise errors (FWE) as indicated for each comparison [25].

Diffusion tensor imaging (DTI) was performed using an echo-planar, spin echo imaging sequence. The following parameters were used: 24 cm field of view (FOV), 128 X 128 matrix size, 27 to 5 mm thick slices acquired interleaved on an axial plane with anterior-posterior phase encoding, four averages, repetition time (TR) = 3,700 ms, echo time (TE) = 93 ms, 1,396 Hz/Px bandwidth, and b-values of 0 and 1,000 s/mm^2^ and 20 gradient directions. A 3D MPRAGE anatomical image was also acquired (256 X 256 matrix, 256 mm FOV, Inversion time (TI) = 900 ms, TR = 1900 ms, TE = 2.2 ms, bandwidth = 2,332 Hz/Px).

White matter fibers for the whole brain were reconstructed for each subject with the DTI Track module of MedINRIA (http://www-sop.inria.fr/asclepios/software/MedINRIA/), using the following settings: fractional anisotropy (FA) = 0.2, smoothness = 20, minimum fiber length = 40 mm, and sampling = 1. All fibers were included in a bundle, for which volume, number of fibers, fiber length, FA, and apparent diffusion coefficient (ADC) were calculated.

### Region of interest analysis

Cortical regions of interest (ROIs) were defined as spheres of 10 mm radius centered on the voxel of highest intensity in each area of activation of interest. The anterior and posterior cingulate were defined using the ‘Talairach Daemon Labels’ feature of Pickatlas 2.4 (http://fmri.wfubmc.edu/software/PickAtlas). The *t* values and mean and standard deviation of intensity were calculated for each ROI using Pickatlas.

## Results

### Subject characteristics

The TD (n = 21), ARND (n = 22) and ADHD (n = 20) groups were well-matched for age and gender distribution (Table 1). Full-scale IQ (FSIQ) was significantly lower in the ARND (range: 48 to 89) group than both the TD (range: 89 to 135) and ADHD (range: 63 to 122) groups, a finding typical of FASD subjects [26]. Socioeconomic status, represented by the average household income in the neighborhood of residence, was significantly lower in the ARND group than the TD group.

### Task performance

Accuracy on both the 0-back and 1-back tasks was high in all three groups (Table 2). On the 1-back task, the TD group performed better than the ARND and ADHD groups who made significantly more errors of commission. Although these differences in accuracy are statistically significant, the number of errors, and thus the number of trials excluded from the fMRI analysis, was not high enough to introduce significant bias into the analysis.

**Table 2:** Working memory task performance

**Parameter**	**TD (n = 21)**		**ARND (n = 22)**		**ADHD (n = 20)**		**Significance**^**a**^	***Post-hoc*tests**
	Mean	Std Dev	Mean	Std Dev	Mean	Std Dev		
Working memory index (WISC)	100.86	12.65	69.62	16.49	89.80	12.28	F = 29.986 *P* <0.001	TD > ARND^b^*P* <0.001
								ADHD > ARND *P* <0.001
Working memory - Parent (BRIEF)	50.38	9.96	75.50	9.09	69.05	7.79	F = 44.320 *P* <0.001	TD < ARND^b^*P* <0.001
								TD < ADHD *P* <0.001
1-back accuracy (%)	94.87	4.25	80.94	12.06	79.79	13.88	F = 12.495 *P* < 0.001	TD > ARND^c^*P* <0.001
								TD > ADHD *P* <0.001
0-back accuracy (%)	95.63	3.48	88.64	8.15	89.46	10.34	F = 5.052 *P* = 0.009	
1-back errors (%)	1.93	2.03	11.42	9.89	11.39	8.08	F = 11.158 *P* <0.001	TD < ARND^c^*P* <0.001
								TD < ADHD *P* <0.001
1-back non-response (%)	3.20	3.26	7.64	6.93	10.21	10.59	F = 4.642 *P* = 0.013	
1-back response time (ms)	538	115	623	192	667	183	F = 3.197 *P* = 0.048	
0-back response time (ms)	740	184	910	330	964	266	F = 3.983 *P* = 0.024	
1-back response variability (ms)	203	78	305	110	336	131	F = 8.669 *P* <0.001	TD < ARND^c^*P* = 0.008
								TD < ADHD *P* = 0.001
0-back response variability (ms)	188	98	275	126	303	123	F = 5.581 *P* = 0.006	

^a^Bonferroni-corrected *P* value for multiple comparisons = 0.005; ^b^Tukey’s HSD test; ^c^Games-Howell test. ADHD, attention deficit/hyperactivity disorder; ARND, alcohol related neurodevelopmental disorder; BRIEF, Behavior Rating Inventory of Executive Function; TD, typically developing; WISC, Wechsler Intelligence Scale for Children.

ARND subjects achieved lower scores than the TD and ADHD groups on the WISC-IV Working Memory Index (WMI) and the BRIEF parental ratings of working memory (WMP, Table 2). Across all subjects, 1-back accuracy was significantly correlated with WMI (Spearman’s ρ = 0.558, *P* <0.001) and WMP (Spearman’s ρ = −0.542, *P* = 0.001), consistent with the assumption that the 1-back task measures working memory. Accuracy for the two tasks was equal for subjects in the TD group (paired *t* = −0.65, *P* = 0.523), whereas accuracy was lower for the more challenging 1-back task in both the ARND (paired *t* = −2.973, *P* = 0.007) and ADHD (paired *t* = −5.271, *P* < 0.001) groups.

There were no significant differences in response times among the groups for either task (Table 2). Subjects in all groups answered faster on the 1-back than the 0-back task (TD: paired *t* = −8.317, *P* < 0.001, ARND: paired *t* = −7.017, *P* < 0.001, ADHD: paired *t* = −7.216, *P* < 0.001). This relationship was expected, as the 1-back task required subjects to hold the response in working memory until the onset of the next trial, at which time subjects likely responded as soon as possible before encoding the response for the next trial.

The variability in response time, measured as the standard deviation of response times across all trials, was significantly higher in the ARND and ADHD groups as compared to the TD group (Table 2). There were no significant differences in response time variability between the tasks for any of the groups.

There was a significant negative correlation between accuracy and response time in the 1-back task for the ADHD group (ρ = −0.606, *P* = 0.005), indicating that subjects who took more time to respond did so with more errors. This relationship was not significant for the TD and ARND groups.

### fMRI results

#### 0-Back task

**Figure 2:**
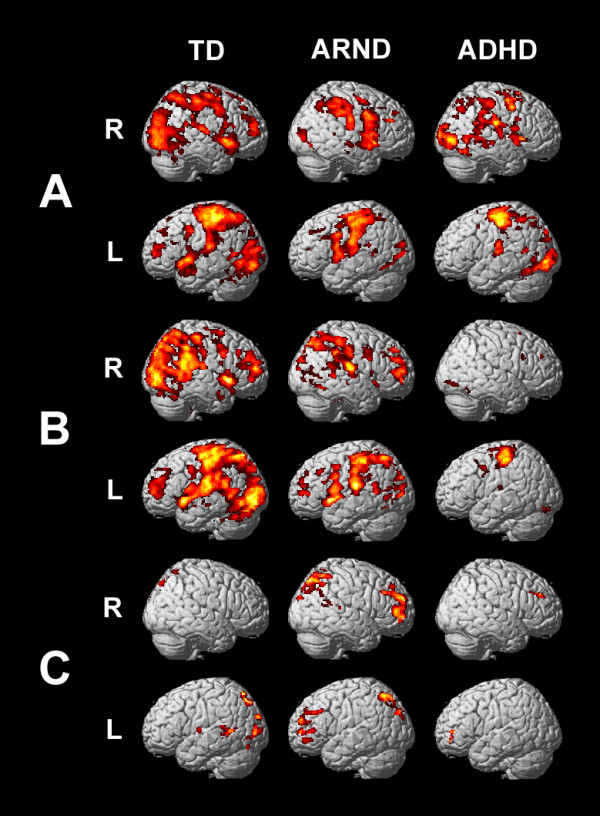
fMRI activation maps for A) the 0-back contrast (FWE = 0.001, corrected for multiple comparisons using cluster size = 10), B) the 1-back contrast (FWE = 0.001, corrected for multiple comparisons using cluster size = 10), and C) the subtractive (1-back minus 0-back) contrast (FWE = 0.05, corrected for multiple comparisons using cluster size = 22). R = right, L = left.

Blood oxygen level dependent (BOLD) activations for the TD group during the 0-back task included bilateral frontal, parietal, temporal, and occipital cortical regions (Figure 2A, corrected *P* < 0.001) consistent with the performance of a visuospatial attention task [27,28]. Activations in the frontal cortex included the dorsolateral prefrontal cortex (DLPFC, Brodmann areas (BAs) 8, 9, 10, and 46), ventrolateral prefrontal cortex (VLPFC, BAs 45 and 47), insula (BA 13), and the supplementary motor area (SMA, BA 6) and pre-SMA. A strong activation was observed in the left motor region (BA 6), corresponding to the subjects’ manual responses. Parietal activations included the precuneus and the superior (BA 7) and inferior (BA 40) parietal lobules. There were strong activations throughout the occipital cortex, including the cuneus and primary (BA 17) and secondary (BAs 18 and 19) visual processing regions. Temporal activations included the bilateral superior (BA 22), left middle (BA 21), and bilateral transverse temporal gyri (BAs 41 and 42), and extended posteriorly to the occipital and fusiform gyrus (BA 37). Other activations included the cingulate (BAs 31 and 32) and right cerebellum. During the 0-back task, the ARND and ADHD groups activated a similar network (Figure 2).A

**Figure 3:**
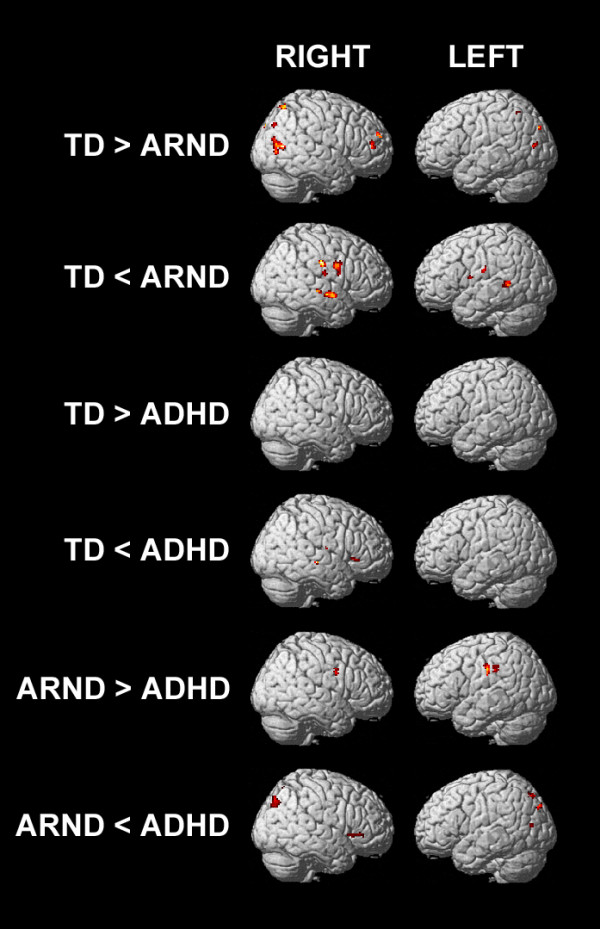
fMRI activation maps for the pairwise comparisons of the 0-back contrast (FWE = 0.0167, corrected for multiple comparisons using cluster size = 12).

Differences between the groups were examined by pairwise one-tailed *t*-tests (Figure 3*P* = 0.0167). The ARND group had lower activation than the TD group in the right parietal cortex (BAs 7 and 40), right inferior (BA 46) and medial frontal (BA 10) gyri, and the anterior cingulate (BA 32), all of which are important in attention processes [28]. The ARND group had greater activation than the TD group in bilateral temporal cortex (BAs 21 and 22), and greater activation than both the TD and ADHD groups in bilateral pre- and postcentral regions, as well as the thalamus. The ADHD group had greater activation of the precuneus and right inferior frontal gyrus (BA 47 and 45), and much lower activation of the caudate, relative to the TD and ARND groups. Activation in the cingulate (BA 24) was weaker in the ADHD group than in the ARND group.

#### 1-Back task

The TD and ARND groups activated a similar network of regions in response to the 1-back task as they did to the 0-back task (Figure 2B), consistent with the extensive overlap of the brain regions recruited in response to attention [28] and working memory [27] tasks. In contrast, the ADHD group had weak activations in all cortical areas during the 1-back task, aside from a robust activation of the motor area (BA 6). That the activation maps of individual ADHD subjects were of similar intensity to those of the TD and ARND groups suggests that the weak activations of the ADHD group as a whole is the result of multiple pathways of activation in response to the 1-back task. It is noteworthy that, when ADHD subjects with comorbid disorders (see Comorbid Disorders below) were excluded from this analysis, a further activation was observed in the left DLPFC (BA 10).

**Figure 4:**
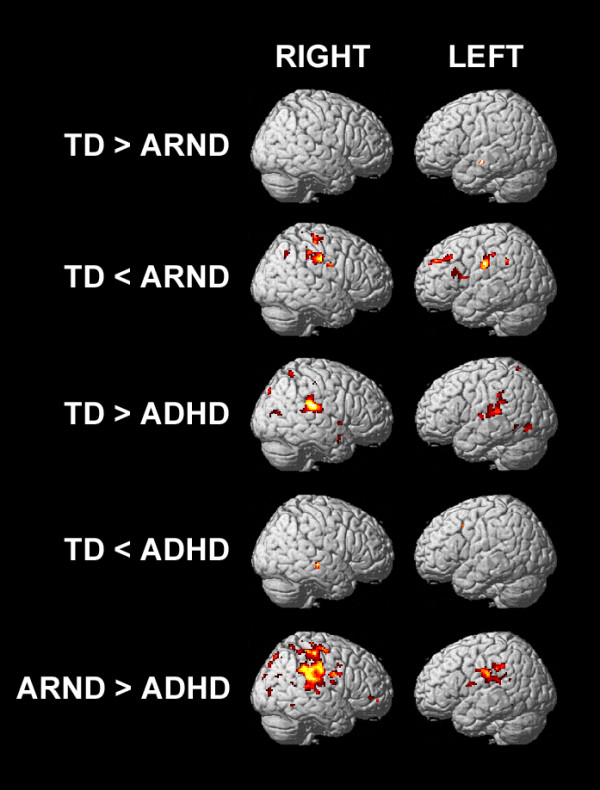
fMRI activation maps for the pairwise comparisons of the 1-back contrast (FWE = 0.0167, corrected for multiple comparisons using cluster size = 12).

Differences between the groups were examined by pairwise one-tailed *t*-tests (Figure 4, *P* = 0.0167). The ARND group had greater activation than the TD group across the left DLPFC (BAs 6, 9, and 10) and left inferior frontal gyrus (BAs 45 and 44), SMA, right-lateralized pre- and postcentral regions, and a region of the cuneus and precuneus extending into the posterior cingulate. The TD group had greater activation than the ARND group in the left superior temporal (BA 22) and lingual (BA 19) gyri.

The ADHD group had weaker activation than the TD group across a broad posterior region including the inferior parietal lobules (BA 40), posterior temporal cortex (BAs 39 and 19), and right anterior temporal gyrus (BA 38), extending through the insula to the inferior frontal gyrus (BA 47). The ADHD group had greater activation than the TD group in the left DLPFC and right middle temporal gyrus.

There were no regions in which the ADHD group had greater activation than the ARND group. The ARND group had greater activation than the ADHD group in bilateral pre- and postcentral regions, right middle frontal gyrus (BAs 10 and 6), and left inferior frontal gyrus (BAs 45 and 44).

#### Subtractive contrast (1-Back minus 0-Back)

The 0-back and 1-back tasks share a number of features, including visual, attention, response selection and motor processes. Subtraction of activation maps from the simpler 0-back task from those of the more complex 1-back task results in a map depicting regions which were activated more strongly during the 1-back task [29]. These activations may be expected to correspond to processes unique to the 1-back task, such as storage and retrieval of data in working memory, and processes which may be more strongly recruited during the 1-back task due to its increased difficulty, such as attention.

In the subtractive contrast, the TD group more strongly activated posterior parietal regions, including the precuneus and the superior parietal lobule (BA 7), than during the 0-back task (Table 3 and Figure 2C), indicating that greater effort was required to complete the 1-back task successfully. Increased activation was also observed across the occipital cortex and in the left posterior temporal cortex (BA 22) and posterior cingulate (BAs 30 and 31). The subtractive contrast of the ARND group (Figure 2C and Table 4) indicated greater recruitment of prefrontal (BAs 8, 9, 44 and 45), pre-SMA and superior posterior parietal cortices, including the precuneus, consistent with increased working memory load. Significantly increased activation was also observed in the bilateral inferior frontal gyri (BA 47) and the cingulate (BAs 31 and 32), as has been observed in a study of children and adults with FASD performing a spatial working memory task [23]. The ADHD group activated very few regions in the subtractive contrast (Figure 2C and Table 5), which were confined to the left inferior frontal and bilateral middle frontal gyri.

**Table 3:** **Regions activated by the TD group during working memory (*P*** **> 0.05)**

**Region**	**Hemisphere**	**Gyrus**	**BA**	**Talairach Coordinates**			**Voxel T**	**Cluster Extent**
				**X**	**Y**	**Z**		
Parietal	Left	Superior Parietal Lobule	7	−12	−67	59	3.22	
	Right	Precuneus	7	2	−74	35	4.39	1,276
		Precuneus	7	2	−66	44	3.59	
		Precuneus	7	10	−55	58	2.14	
		Superior Parietal Lobule	7	18	−57	62	2.53	107
		Superior Parietal Lobule	7	14	−61	55	2.34	
Occipital	Left	Cuneus	19	−22	−88	30	3.37	91
		Cuneus	30	−16	−68	7	3.4	640
		Fusiform	19	−32	−76	−10	2.22	22
		Lingual	18	−12	−70	−7	3.06	
		Lingual	18	−10	−82	−6	2.13	48
		Middle Occipital	19	−36	−89	10	4.3	146
	Right	Cuneus	17	10	−79	11	2.55	
		Lingual	17	18	−89	−1	2.15	
		Lingual	18	20	−78	−5	2.36	68
Temporal	Left	Middle Temporal	22	−48	−43	2	2.73	201
		Middle Temporal	22	−59	−47	2	2.52	
		Superior Temporal	22	−55	−7	8	2.82	29
		Superior Temporal	39	−48	−52	10	2.66	
Limbic	Left	Posterior Cingulate	31	−22	−65	14	2.96	
	Right	Parahippocampal	28	20	−26	−7	2.25	32
		Posterior Cingulate	30	18	−68	7	2.7	
		Posterior Cingulate	31	12	−65	16	3.02	262
Cerebellum	Left	Culmen		−4	−36	−18	3.02	
Midbrain	Right	Red Nucleus		4	−25	−4	2.26	35
Brain Stem				0	−30	−22	3.37	127

TD, typically developing.

**Table 4:** **Regions activated by the ARND group during working memory (*P*** **> 0.05)**

**Region**	**Hemisphere**	**Gyrus**	**BA**	**Talairach Coordinates**			**Voxel T**	**Cluster Extent**
				**X**	**Y**	**Z**		
Frontal	Left	Inferior Frontal	47	−32	23	−6	2.06	25
		Medial Frontal	8	0	26	47	2.11	
		Medial Frontal	9	−20	43	14	2.83	
		Middle Frontal	10	−28	51	20	3.32	549
		Middle Frontal	11	−40	48	−9	2.94	75
		Middle Frontal	47	−42	40	−9	2.23	
		Middle Frontal	9	−38	31	28	2.66	
		Middle Frontal	11	−24	47	1	2.68	339
		Superior Frontal	8	0	16	49	2.6	70
	Right	Middle Frontal	10	28	52	21	3.69	
		Middle Frontal	9	32	43	38	3.71	
		Superior Frontal	10	32	56	−3	3.89	1,513
Parietal	Left	Precuneus	31	−8	−71	24	1.88	44
		Superior Parietal Lobule	7	−12	−61	55	4.41	3,179
		Superior Parietal Lobule	7	−18	−67	55	3.61	
	Right	Precuneus	7	2	−70	46	3.5	
		Supramarginal	40	57	−57	30	2.3	29
Occipital	Left	Cuneus	18	−16	−71	20	1.83	
	Right	Cuneus	19	22	−86	28	3.03	67
Temporal	Right	Superior Temporal	13	59	−44	19	1.96	
		Superior Temporal	22	61	−54	17	2.49	34
		Superior Temporal	41	50	−32	13	1.88	23
Limbic	Left	Anterior Cingulate	32	−12	35	4	2.47	
		Anterior Cingulate	32	−20	37	4	2.53	
		Cingulate	32	0	27	34	2.69	67
Midbrain	Right	Red Nucleus		2	−16	−4	3.48	68
Sub-lobar	Left	Lentiform Nucleus		−16	−2	2	2.65	29

ARND, alcohol related neurodevelopmental disorder.

**Table 5:** **Regions activated by the ADHD group during working memory (*P*** **> 0.05)**

**Region**	**Hemisphere**	**Gyrus**	**BA**	**Talairach Coordinates**			**Voxel T**	**Cluster Extent**
				**X**	**Y**	**Z**		
Frontal	Left	Inferior Frontal	10	−44	45	3	2.07	
		Inferior Frontal	10	−44	46	−4	2.16	38
		Middle Frontal	11	−38	48	−9	2.09	
	Right	Middle Frontal	46	46	42	26	2.81	45
		Middle Frontal	9	51	31	30	2.22	

ADHD, attention deficit/hyperactivity disorder.

**Figure 5:**
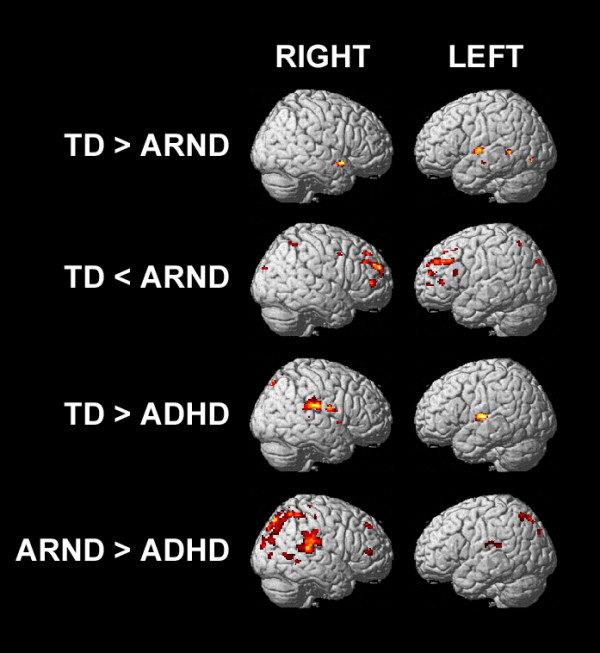
fMRI activation maps for the pairwise comparisons of the subtractive contrast (FWE = 0.0167, corrected for multiple comparisons using cluster size = 12).

Differences between the groups were examined by pairwise one-tailed *t*-tests (Figure 5, *P* = 0.0167). The TD group had greater activation than the ARND group (Table 6) in the bilateral temporal cortex, whereas the ARND group had greater activation than the TD group (Table 7) throughout the DLPFC, the left inferior frontal gyrus, pre-SMA, anterior cingulate (BA 32), and the precuneus extending to the bilateral superior parietal lobules. There were no regions in which the ADHD group had greater activation than either the TD or the ARND groups. Both the TD (Table 8) and ARND (Table 9) groups had greater activation than the ADHD group in the middle and posterior cingulate, bilateral inferior parietal lobules, and precuneus.

**Table 6:** **Regions in which the TD group had greater activation than the ARND group in the subtractive contrast (*P*** **> 0.0167)**

**Region**	**Hemisphere**	**Gyrus**	**BA**	**Talairach Coordinates**			**Voxel T**	**Cluster Extent**
				**X**	**Y**	**Z**		
Sub-lobar	Left	Thalamus		0	−11	19	2.53	26
	Right	Insula		40	−10	−1	2.74	21
Limbic	Left	Parahippocampal	35	−16	−29	−5	3.17	36
	Right	Amygdala		28	1	−20	2.90	18
Occipital	Left	Inferior Occipital	19	−36	−74	−3	2.58	38
		Lingual	19	−18	−66	3	2.97	34
		Lingual		−30	−74	2	2.44	
Temporal	Left	Middle Temporal	22	−48	−44	4	2.79	38
		Sub-Gyral	21	−44	−14	−9	2.43	21
		Superior Temporal	22	−57	−8	4	3.44	63
	Right	Superior Temporal	38	46	7	−10	3.40	69
Cerebellum	Right	Culmen		12	−33	−7	3.22	37
		Culmen		16	−41	−8	2.34	
		Culmen		14	−32	−22	2.55	19
Midbrain	Left	Red Nucleus		−2	−20	−14	2.92	29
		Brain Stem		0	−30	−24	2.57	20

ARND, alcohol related neurodevelopmental disorder; TD, typically developing.

**Table 7:** **Regions in which the ARND group had greater activation than the TD group in the subtractive contrast (*P*** **> 0.0167)**

**Region**	**Hemisphere**	**Gyrus**	**BA**	**Talairach Coordinates**			**Voxel T**	**Cluster Extent**
				**X**	**Y**	**Z**		
Frontal	Left	Inferior Frontal	45	−50	37	6	2.76	28
		Inferior Frontal	45	−55	20	16	2.58	22
		Medial Frontal	8	−2	41	38	2.61	14
		Middle Frontal	8	−18	31	41	2.46	33
		Middle Frontal	8	−36	22	45	2.46	12
		Sub-Gyral		−22	45	1	2.98	116
		Superior Frontal		−28	52	1	2.45	
	Right	Inferior Frontal	10	44	45	3	3.10	58
		Middle Frontal	10	28	52	21	3.25	
		Middle Frontal	8	34	41	38	3.01	29
		Middle Frontal	9	50	8	38	2.56	13
		Paracentral Lobule	5	8	−36	55	2.31	15
		Sub-Gyral	3	18	−32	55	2.30	
		Superior Frontal	9	10	55	19	3.36	
		Superior Frontal	9	20	43	38	2.59	26
		Superior Frontal	8	2	20	47	3.27	107
	Right	Superior Frontal	9	16	39	33	2.28	
Sub-lobar	Left	Lateral Globus Pallidus		−14	0	0	2.44	14
Limbic	Left	Anterior Cingulate	32	−10	34	20	3.48	1,318
		Anterior Cingulate	32	−20	37	4	2.58	
		Anterior Cingulate	32	−8	42	−7	2.70	46
	Right	Anterior Cingulate	32	6	33	−5	2.68	15
Parietal	Left	Precuneus	19	−28	−82	35	2.70	23
		Precuneus	7	−2	−64	49	2.65	42
		Precuneus	7	0	−72	46	2.28	
		Superior Parietal Lobule	7	−28	−57	54	2.42	15
	Right	Inferior Parietal Lobule	40	40	−50	54	2.56	31
Occipital	Right	Cuneus	19	22	−86	28	2.72	30
Midbrain	Right	Red Nucleus		2	−16	−4	3.64	44

ARND, alcohol related neurodevelopmental disorder; TD, typically developing.

**Table 8:** **Regions in which the TD group had greater activation than the ADHD group in the subtractive contrast (*P*>0.0167)**

**Region**	**Hemisphere**	**Gyrus**	**BA**	**Talairach Coordinates**			**Voxel T**	**Cluster Extent**
				**X**	**Y**	**Z**		
Frontal	Left	Paracentral Lobule	5	0	−40	54	2.80	60
		Precentral	43	−55	−9	10	3.12	153
	Right	Inferior Frontal	44	57	5	15	2.30	
Sub-lobar	Left	Insula	13	−44	−17	6	2.54	
	Right	Insula	13	40	−22	−4	2.56	
		Insula	13	42	−18	25	2.42	44
		Insula	13	38	−24	20	2.39	
		Insula	13	48	6	2	2.36	24
Limbic	Left	Cingulate	31	−12	−43	28	2.61	30
		Cingulate	31	−16	−46	19	2.49	21
		Posterior Cingulate	30	−10	−62	12	3.25	
	Right	Parahippocampal	28	22	−28	−9	3.02	137
Parietal	Right	Inferior Parietal Lobule	40	63	−28	24	3.66	
		Inferior Parietal Lobule	40	59	−24	31	2.38	
		Postcentral	40	63	−20	21	4.02	205
		Postcentral	43	59	−5	17	3.02	57
		Precuneus	7	8	−57	58	2.72	47
		Precuneus	7	16	−73	50	2.66	28
Temporal	Right	Fusiform	37	30	−40	−13	3.15	70
		Hippocampus		30	−24	−11	2.92	
		Superior Temporal	41	48	−33	9	2.67	22
Cerebellum	Left	Culmen		−10	−53	−4	3.29	
		Culmen		−22	−36	−12	2.91	131
		Culmen		−16	−40	−17	2.78	
		Culmen of Vermis		−4	−62	1	3.71	433

ADHD, attention deficit/hyperactivity disorder; TD, typically developing.

**Table 9:** **Regions in which the ARND group had greater activation than the ADHD group in the subtractive contrast (*P*** **> 0.0167)**

**Region**	**Hemisphere**	**Gyrus**	**BA**	**Talairach Coordinates**			**Voxel T**	**Cluster Extent**
				**X**	**Y**	**Z**		
Frontal	Right	Inferior Frontal	45	50	37	7	2.52	20
		Inferior Frontal	10	46	43	3	2.19	
		Middle Frontal	10	38	43	7	2.34	16
		Middle Frontal	9	32	45	36	2.61	40
		Middle Frontal	8	26	39	39	2.47	
		Paracentral Lobule	5	6	−38	53	3.37	
		Precentral	4	30	−23	51	2.55	27
Sub-lobar	Left	Thalamus		−2	−23	12	2.66	37
		Thalamus		−4	−22	20	2.32	
	Right	Claustrum		38	−22	−4	2.43	15
		Insula	13	30	−32	22	2.41	20
		Thalamus		16	−35	5	2.51	16
Limbic	Left	Cingulate	31	−16	−44	22	2.32	15
		Posterior Cingulate	30	−10	−62	12	3.49	460
		Posterior Cingulate	29	−8	−38	17	2.58	15
		Posterior Cingulate	23	−10	−28	24	2.48	
		Posterior Cingulate	23	−2	−34	24	2.40	46
	Right	Cingulate	24	4	15	27	2.66	27
		Cingulate	31	12	−38	26	2.33	15
		Parahippocampal	30	16	−45	1	2.60	20
Parietal	Left	Postcentral	40	−61	−24	21	2.51	
		Precuneus	7	−12	−66	48	3.42	
		Precuneus	19	−32	−80	37	2.37	14
	Right	Inferior Parietal Lobule	40	63	−30	24	3.72	712
		Postcentral	40	65	−20	21	3.61	
		Postcentral	3	20	−28	62	2.99	43
		Precuneus	7	16	−73	48	3.49	3,072
Occipital	Left	Cuneus	18	−12	−71	16	2.61	
		Cuneus	19	−14	−82	37	2.40	16
		Lingual	18	−8	−58	5	3.06	
	Right	Middle Occipital	19	42	−81	10	2.58	19
Temporal	Left	Fusiform	20	−30	−36	−15	2.60	55
		Superior Temporal	42	−61	−32	18	2.66	41
	Right	Fusiform	37	40	−47	−9	2.92	26
		Fusiform	20	30	−40	−15	2.80	20
		Middle Temporal	21	59	−47	1	2.61	19
		Middle Temporal	37	46	−56	5	2.40	49
		Superior Temporal	41	50	−30	13	3.07	
Cerebellum	Left	Culmen		−20	−32	−14	2.48	

ADHD, attention deficit/hyperactivity disorder; ARND, alcohol related neurodevelopmental disorder.

### Age

Since age was well-matched among the groups (Table 1), a between-groups effect of age was not expected. However, the age range in this study corresponded to a broad developmental period during which brain development relevant to working memory, especially in the frontal lobes, is ongoing [30] and could introduce the possibility of within-groups effects of age. Calculation of the *Z* score of 1-back accuracy using age as the grouping variable did not modulate the superior accuracy of the TD group relative to the ADHD and ARND groups (TD-ADHD *Z* = −4.21, *P* < 0.0167; TD-ARND *Z* = −4.49, *P* < 0.0167; ADHD-ARND *Z* = 0.56, *P* >0.0167), as was observed in an earlier fMRI study of FASD children performing a working memory task [21]. The activation map for the 1-0-back null hypothesis was unchanged by the inclusion of age as a covariate (data not shown), indicating that subject age was not a significant contributor to the activations observed.

### IQ

Since the mean FSIQ for the ARND group was lower than that of the TD group (Table 1), it was important to assess the contribution of FSIQ to the differences among the groups. It was also important to ascertain that the ARND subjects with the lowest FSIQ scores were not exerting undue influence on the results of the ARND group.

Three subjects in the ARND group had FSIQ scores below 70, the threshold for diagnosis of cognitive impairment. On the 1-back task, only one of these subjects had an accuracy score more than one standard deviation below average for the group and two had faster response times and lower response time variability than the averages of the group. Thus, it cannot be concluded that these subjects were impaired in their performance on the working memory task relative to the other members of the ARND group. To determine whether these subjects were skewing the fMRI results, the subtractive contrast including all subjects in the ARND group (Figure 2C, Table 4) was compared to that excluding the low-IQ subjects. There were no significant differences (*t*-test, *P* = 0.05), indicating that these subjects were not outliers.

To assess the possibility that the wide range of FSIQ among the ARND group was obscuring common activations in their fMRI data, the subtractive analysis was repeated for this group using FSIQ as a covariate. The resulting activation map was unchanged. Since including FSIQ as a covariate in the single-group analysis of the ARND group had no effect on the activation map for the subtractive contrast, it is likely that any effects of IQ are mediated between groups, rather than within groups.

### Comorbid disorders

As described in the Methods section, a number of subjects in the ADHD and ARND groups had comorbid disorders, a finding typical of ARND [1]. None of these subjects had accuracy scores or response times more than two standard deviations from the means of their respective groups, and excluding these subjects from the analysis did not significantly affect the task performance results given in Table 2. Thus, these subjects were not outliers in behavioral terms. To determine whether these subjects were skewing the fMRI results of their respective groups, the subtractive contrasts of the ARND and ADHD groups (Figure 2C, Tables 4 and 5) were compared to those excluding subjects with comorbid disorders. There were no significant differences (*t*-test, *P* = 0.05), indicating that these subjects were not outliers. The pairwise comparisons of the three groups was also repeated on the subtractive contrast, excluding the subjects with comorbid disorders as well as the three ARND subjects with FSIQ scores below 70. The differences among the groups were essentially identical to the results using the whole groups (Figure 4, Tables 6,7,8,9), taking into account the loss of power resulting from the smaller size of the ARND (n = 16) and ADHD (n = 15) groups. Thus, there is no empirical basis for the exclusion of these subjects from the fMRI analysis.

### ARND subjects with diagnoses of ADHD

**Figure 6:**
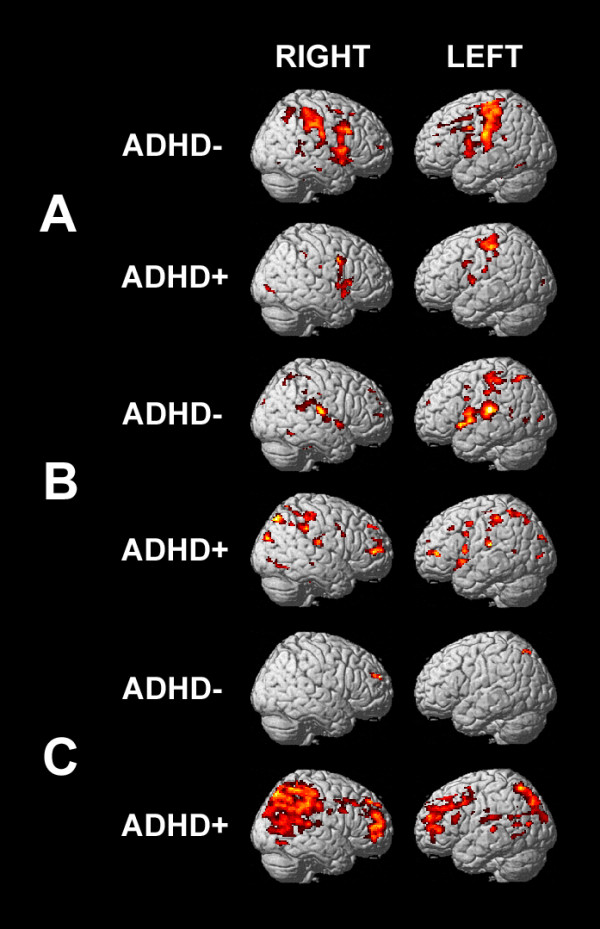
**fMRI activation maps for ARND subjects with and without diagnoses of ADHD.** FWE = 0.001 for **A**) the 0-back and **B**) 1-back contrasts (corrected for multiple comparisons using cluster size = 10), and FWE = 0.05 for **C**) the subtractive contrast (corrected for multiple comparisons using cluster size = 22). ADHD, attention deficit/hyperactivity disorder; ARND, alcohol related neurodevelopmental disorder; fMRI, functional MRI.

ADHD symptomatology is common among children with FASD [26]. Indeed, 11 of the 22 children in the ARND group also had a diagnosis of ADHD. During the 0-back task, the ARND/ADHD + subgroup activated pre- and postcentral, ventrolateral frontal, and anterior temporal regions (Figure 6A). Children in the ARND/ADHD- subgroup activated these regions significantly more strongly than the ARND/ADHD + subgroup, as well as dorsolateral frontal and parietal regions. During the 1-back task, the ARND/ADHD + subgroup had strong activations across the DLPFC (BAs 9 and 10), pre-SMA, and lateral posterior parietal cortex (left BA 40 and right BA 39, Figure 6B), resulting in strong activations across these regions in the subtractive contrast (Figure 6C), which corresponds to working memory load. In contrast, the ARND/ADHD- subgroup had weaker parietal activations and stronger bilateral temporal activations during the 1-back task, resulting in activations in the subtractive contrast much weaker than that of the ARND/ADHD + subgroup. It is noteworthy that there were no significant differences in subject characteristics or task performance (data for the ARND group as a whole are given in Tables 1 and 2, respectively) among the two subsets of the ARND group. However, all four subjects with comorbid disorders other than ADHD were included in the ARND/ADHD + subgroup.

To directly evaluate differences between the ADHD group and the ARND subgroups, pairwise comparisons were made of the subtractive contrast (*P* = 0.0125, cluster size = 10). As was the case for the entire ARND group, there were no regions in which either the ARND/ADHD + or the ARND/ADHD- group had weaker activation than the ADHD group. The ARND/ADHD- group activated the right inferior parietal lobule and precuneus, extending into the bilateral superior parietal lobules significantly more than the ADHD group, similar to the comparison of the entire ARND group (Figure 4), whereas the ARND/ADHD + group had greater activation across a much broader region encompassing pre- and post-central and temporal cortices.

The regions in which each ARND subgroup had weaker activation than the TD group were generally similar to the comparison of the entire ARND group (Figure 4). The subgroups differed markedly, however, in the regions of greater activation relative to the TD group: the ARND/ADHD + subgroup had greater activation across a broad region of frontal, parietal, and superior temporal regions, whereas the ARND/ADHD- subgroup had greater activation in a small region of the right DLPFC and the right superior parietal lobule.

### fMRI ROI analysis

**Figure 7:**
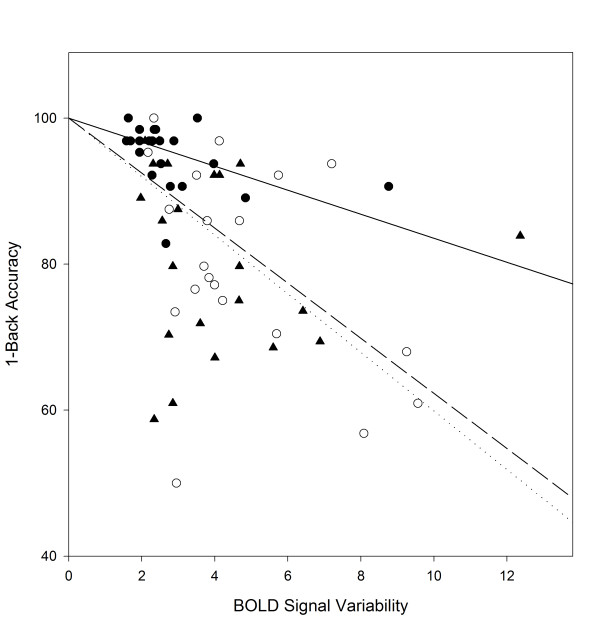
**Relationship of intrasubject variability and performance.** Data for TD (·), ARND (○), and ADHD (▴) were fitted to linear equations of the form y = 100 – mx. ADHD, attention deficit/hyperactivity disorder; ARND, alcohol related neurodevelopmental disorder; TD, typically developing.

Regions of significant activation common to all groups in the subtractive contrast were identified and those in cortical regions known to be of importance in attention and working memory tasks were selected for ROI analysis (Table 10). No group-wise differences were observed in the intensities or *t* values of the chosen ROIs. In contrast, the average standard deviation, which measures intra-subject variability in the BOLD signal, was highly correlated to several task performance measures, the strongest of which was accuracy on the 1-back task. Since the standard deviations of the eight ROIs were highly correlated (data not shown), they were averaged into a single value for each subject, which was significantly different among the groups (*F* = 4.278, *P* = 0.018), indicating differences among the groups in the within-subject variability of the fMRI activations. *Post-hoc* tests revealed that the average standard deviation for the TD group was significantly lower than that of the ADHD group. The linear regression analysis for each group (Figure 7) demonstrates that, for the ARND and ADHD groups, intra-subject variability is more strongly correlated to 1-back accuracy than for the TD group, with higher accuracy corresponding to low BOLD signal variability. Intercept terms were not significant and were thus dropped from the equations.

**Table 10:** Definitions of ROIs

**ROI**	**BA**	**Shape**^**a**^	**Talairach Coordinates**		
			**X**	**Y**	**Z**
L Middle frontal	10	Sphere	−32	51	1
R Middle frontal	10	Sphere	26	52	21
R Insula	13	Sphere	32	3	18
L Inferior parietal	40	Sphere	−61	−31	37
L Middle temporal	21	Sphere	−51	1	−19
R Inferior temporal	37	Sphere	55	−58	−4
Anterior cingulate		Cortical surface			
Posterior cingulate		Cortical surface			

^a^Spheres were defined with radius 10 mm, using the Talairach coordinates as the centers. The Pickatlas TD labels were used to define the cingulate regions. ROI, region of interest; TD, typically developing.

### Tractography

Fiber tracking was performed on the whole brain white matter. Fiber tracking parameters (total fiber volume, number of fibers, maximum fiber length, mean fiber length, and mean FA) were significantly negatively correlated with task errors (1-back errors of choice) across all subjects (*P* <0.01), indicating that subjects with greater white matter integrity performed better on the 1-back task. One-way ANOVAs on each of the fiber tracking parameters indicated significant differences among the groups for total fiber volume (F = 6.391, *P* = 0.003) and number of fibers (F = 6.239, *P* = 0.004). *Post-hoc* tests revealed that the TD group had higher volume (Tukey HSD *P* = 0.002) and number of fibers (*P* = 0.003) than the ARND group. There were no differences in any of these measures between the ADHD group and either the TD or the ARND group.

## Discussion

### TD group

Brain activation by the TD group was consistent with the performance of a spatial working memory task. Activations during the 1-back task included the expected dorsal and ventral attentional networks as defined by Corbetta and Shulman [28], which represent top-down and bottom-up attention, respectively. Bilateral activation of the SMA, which has been associated with visual attention [31], motor readiness [32] and rehearsal processes [33,34] was also observed. Although spatial working memory has frequently been observed to be right-lateralized [29,34,35], lateralization of activations was not observed in response to the n-back task (Figure 2), with the exception of the large activation in the left motor area corresponding to the subjects’ manual responses.

Activations were observed in the DLPFC, VLPFC, and superior frontal regions (Figure 2). While these activations are consistent with the demands of working memory [27], the TD group did not further activate these regions in the 1-back relative to the 0-back task (Figure 2C), perhaps because the task was designed with a relatively low working memory load to ensure high accuracy scores from all subjects. The strong activation in the bilateral DLPFC (BAs 9, 10 and 46) expected for a spatial working memory task [32] has been associated with monitoring and manipulating information [36] and response selection [37]. These areas have also been associated with holding spatial information on-line, [38]. The VLPFC (BAs 44, 45 and 47) is thought to play a role in comparison of stimuli and response selection [39] and in the detection of unexpected stimuli at searched locations [28].

Broad bilateral activation was observed in the posterior parietal cortex, including the superior and inferior parietal lobules and the precuneus (Figure 2A and B). The 1-back task elicited enhanced medial activation (BA 7) consistent with the putative function of the parietal cortex as a storage buffer for working memory [40].

The robust activation of the occipital cortex indicates engagement of secondary visual processes, likely including the processing of object features and spatial location. The cuneus was also activated, as has been observed in a passive visual task [41]. Activations across the occipital region were enhanced during the 1-back task (Figure 2C and Table 3), possibly consistent with the increased visual scanning during performance of this task compared to the simpler 0-back task.

The anterior cingulate and posterior cingulate (BAs 32 and 31) were activated by the 0-back task, and further activations in the posterior regions (BAs 30 and 31) were observed during the 1-back task, consistent with its putative roles in attention [42], error detection, and conflict monitoring [32,35].

### ARND group

#### Spatial working memory deficit in children with ARND

The ARND group achieved lower accuracy and had greater response time variability on the 1-back task as compared to the TD group (Table 2). These findings were accompanied by significant activations in the prefrontal and posterior parietal cortical regions, the pre-SMA, and the anterior cingulate in the subtractive contrast (Figure 2C and Table 4), suggesting that they employed greater cognitive effort to complete the working memory component of the task.

The ARND group differed from the TD group in terms of IQ and socioeconomic status (Table 1), the presence of comorbid disorders, and medication use, all of which may be expected in a population prenatally exposed to alcohol. Equalization of these differences, whether statistically or by excluding some subjects, would necessarily skew the composition of the group, limiting the generalizability of the results. However, re-analysis of the task performance and fMRI results excluding the subjects with low IQ and comorbid disorders other than ADHD yielded results comparable to those obtained when including all subjects. Thus, while it may be hypothesized that these factors contribute to the presentation of ARND in children, this study neither supports nor refutes these hypotheses.

The TD group activated bilateral prefrontal and posterior parietal regions in response to the 0-back task, the cognitive demands of which are primarily attentional, whereas the ARND group did so only weakly (Figure 2A). This difference was significant in the right DLPFC and bilateral parietal regions (Figure 3). In contrast, the ARND group activated these regions significantly more strongly than the TD group during the 1-back task (Figure 4) and in the subtractive contrast (Figure 5), suggesting that addition of working memory load (for which brain activations overlap considerably with that for attentional networks [27,28]) taxed the data manipulation capacity of ARND subjects. It is also possible that prefrontal and posterior parietal activations by the ARND group were more sensitive to task difficulty, resulting in weak activation to the easier 0-back task and strong activation to the more challenging 1-back task relative to the TD group. Strong prefrontal activation by subjects with FASD has been observed in working memory [21,23], response inhibition [43], and verbal learning tasks [44], and has been attributed to both compensation for inefficient processing elsewhere in the brain and delayed maturation of brain pathways in this group.

For the ARND group, there was a significant negative correlation between the percentage of trials to which subjects did not respond and brain activations in the subtractive contrast which encompassed a broad region including the DLPFC, lateral pre- and postcentral and parietal regions (data not shown). This relationship is consistent with greater activation of these regions as a compensatory strategy to account for inefficient processing. Since there was no corresponding correlation with the percentage of trials to which the ARND subjects responded incorrectly, these increases in brain activation may represent improved attention, rather than storage or retrieval capacity.

Fiber tracking indicated lower total fiber volume and fewer fibers in the ARND group relative to the TD group, indicating that prenatal alcohol exposure had a specific effect on white matter, as has been previously reported [2,45,46]. There was a significant positive correlation between fiber tracking parameters and errors of choice on the 1-back task. It is tempting to speculate a causal relationship between white matter integrity and task performance, which may partially explain the differences in brain activation and deficit in performance by the ARND group on the 1-back task.

#### Secondary visual processing deficit in children with ARND

Significantly greater functional activation was observed in the occipital regions, which are responsible for primary and secondary visual processing, in the TD group than the ARND group during the 0-back task (Figure 3), suggesting either a deficit in visual perception or inefficient visual processing pathways in subjects diagnosed with ARND. Since this decreased activation did not affect performance on the 0-back task (Table 2), subjects must have compensated for this deficit during this simpler task. In the subtractive contrast, which isolates the regions more strongly activated during the working memory component of the 1-back task, the TD group strongly activated the occipital cortex, whereas the ARND group did not (Figures 2C and 5). Since the ARND group performed significantly worse than the TD group on the 1-back task, it may be that ARND subjects were unable to fully compensate for the deficit in visual processing when working memory was involved. This observation is in contrast with an earlier spatial working memory study, in which children with FASD strongly activated this region [21]. In that study, the FASD and control groups had equal accuracy and response times, suggesting the possibility that equal performance might be dependent on sufficient activation of secondary visual regions. Visuospatial cognitive deficits have been repeatedly identified among children with FASD (reviewed in [26,47]). Poor object or location recognition, or a lower capacity to maintain visual images after disappearance of the stimulus [29,48] may, in part, underlie the worse performance of the ARND group on spatial working memory tasks and may place additional demands on the cortical regions responsible for processing downstream events (Figure 2, [21]).

### ADHD group

#### Spatial working memory deficit in children with ADHD

While activations by the ADHD group during the 0-back task were generally similar to those of the TD and ARND groups (Figures 2A and 3), the very weak activation in response to the 1-back task was unique to this group (Figure 2B). Although weak activation by ADHD subjects in response to increasing difficulty has been observed during other working memory tasks [49-51], it is unlikely that task difficulty was sufficient to account for the loss of activation by the ADHD group, especially since their performance did not differ from that of the ARND group (Table 2). It is important to note that both BOLD signal variability (Figure 7) and response time variability (Table 2) were similar between the ARND and ADHD groups. Thus, despite its known importance in explaining the behavior of ADHD subjects, intra-subject variability alone cannot explain the different fMRI activations between these clinical groups.

An earlier study [51] found that children with ADHD had greater activation during the baseline (control) task relative to the working memory task, as shown in the present study (Figure 2). It was concluded that children with ADHD did not have regions of specialized brain activity for working memory [51]. Another possible explanation for the observed differences in cognitive events and behavior is that ADHD subjects may differ from the TD and ARND groups in temporal order judgments [19], an executive process which strongly engages the mid-DLPFC regions (BAs 9 and 46) which were weakly activated by this group [52]. Visual examination of the single-subject activation maps for the 1-back task indicated that they were of similar intensity to those of the 0-back task, and to those of other groups, suggesting that the weak activation by the ADHD group as a whole was due to the absence of a network common to all subjects, as opposed to weaker activations by individual ADHD subjects.

### ARND versus ADHD

The ARND and ADHD groups both performed similarly to the TD group on the 0-back task and worse than the TD group on the 1-back task (Table 2). Both groups had higher intra-subject variability than the TD group as measured by response time variability and BOLD signal variability. Despite similarly impaired performance between the two clinical groups, fMRI results indicate that this outward similarity in behavior may be the result of different cognitive events. The ARND group strongly activated a broad network of cortical regions in response to the 1-back task (Figure 2), consistent with increased effort allocation to compensate for less efficient cognition. The ADHD group, in contrast, weakly activated all cortical regions relative to the TD group during the 1-back task, consistent with a failure to modulate brain activity in response to task demands. Thus, on this relatively easy working memory task, children with ARND and ADHD recruit cognitive resources differently, despite similar resource recruitment on the 0-back task, which has no working memory component.

Subjects diagnosed with ARND and ADHD differed in the nature of their comorbid disorders (see Methods), with learning disabilities being more common in the latter group. While it is not possible to assess the contribution of this difference to the results, it is noteworthy that exclusion of subjects with comorbid disorders (other than ADHD on the ARND group) had essentially no effect. ARND and ADHD subjects also differed in their use of non-stimulant medications (see Methods), the most commonly prescribed of which was risperidone, which has been shown to affect behavior, fMRI activations, and brain morphology in patients with disorders other than ARND [53]. Stimulant medication, use of which did not differ between the ARND and ADHD groups, may, in addition to modulating behavior, affect brain morphology of children with ADHD [54,55]. The conclusions of this study are relevant to typical samples of children taken from the ARND and ADHD populations, who differ in medication use, comorbid conditions, and intellectual functioning.

The ADHD symptomatology within the ARND group may differ from that of the ADHD group. While the ADHD group did not activate the expected working memory networks during the 1-back task (Figure 2B), it was during the 0-back task that the ARND/ADHD + group had low overall activation levels (Figure 6), indicative of a deficit in aspects of the task not requiring working memory, such as attention. Pairwise comparison of the subtractive contrast of the ADHD and ARND/ADHD + groups demonstrated much stronger activation by the latter in all brain regions of known importance to working memory, consistent with a broadly different approach to the task despite similar performance. Qualitative differences in ADHD symptoms between subjects with and without fetal alcohol exposure have been described, but consensus has not yet been reached. One study [12] found that children with FASD performed worse on tests of their ability to encode new information, and to shift attention from one set of rules to another, whereas children with ADHD performed worse on tests of their ability to focus and sustain their attention. However, another study of attention did not identify differences in attention between groups of children with FASD and ADHD [10] and it has been reported that ADHD comorbidity in FASD is associated only with decreased scores on tests of attention, leading the authors to the conclusion that the two disorders are independent of one another [56]. A quite different interpretation has been advanced in a study of verbal memory [13], in which children with FASD had difficulty encoding new information, whereas children with ADHD had difficulty retrieving learned information.

## Conclusions

The present results provide a direct comparison of fMRI activation during a spatial working memory task, in children diagnosed with ARND and those diagnosed with ADHD. Significant increases in the 1-back minus 0-back contrast were noted in the frontal and parietal regions in the ARND group as compared with both the ADHD group and TD controls. This was associated with reduced levels of accuracy and increased response time variability in the ARND group. This activation may reflect recruitment of brain regions associated with the need for ARND subjects to exert greater effort to compensate for the increased difficulty of the 1-back task.

When comparing subgroups of ARND with and without diagnosis of ADHD, the decreased activity observed in the ARND/ADHD + group compared to the ARND/ADHD- group during the 0-back task may indicate a deficit in attentional visuoperceptual processing, since this task made minimal demand on working memory. By contrast, children diagnosed with ADHD weakly activated most cortical regions compared to the TD and ARND groups during the working memory task (1-back and 1-back minus 0-back contrasts). This finding supports the hypothesis that there are differences between the ARND and ADHD groups in terms of the way they recruit cortical regions in support of short term memory for spatial location.

White matter tract integrity in the ARND group compared to the TD group was negatively correlated both with accuracy during the 1-back task and with IQ. By contrast, no differences were observed in the white matter tract parameters between the ADHD group and either the TD or the ARND group. Together with the fMRI results, this suggests that anomalies in task-related cortical activation in the ARND group may reflect pathology in fiber tract development arising from fetal alcohol toxicity, and offer a means to discriminate between subjects diagnosed with ARND and those diagnosed with ADHD.

## Abbreviations

AC, Anterior commissure; ADHD, Attention Deficit Hyperactivity Disorder; ANCOVA, Analysis of covariance; ANOVA, Analysis of variance; ARND, Alcohol-Related Neurodevelopmental Disorder; ASI, Adolescent Symptom Inventory; BA, Brodmann area; BOLD, Blood-oxygen level dependent; BRIEF, Behavior Rating Inventory of Executive Function; CADEC, Clinic for Alcohol and Drug Exposed Children; CNS, Central nervous system; CPT, Continuous Performance Test; CSI, Child Symptom Inventory; DLPFC, Dorsolateral prefrontal cortex; DTI, Diffusion tensor imaging; EPI, Echo-planar imaging; FA, Fractional anisotropy; FAS, Fetal Alcohol Syndrome; FASD, Fetal Alcohol Spectrum Disorder; fMRI, Functional magnetic resonance imaging; FSIQ, Full-scale intelligence quotient; FWE, Family-wise error; HSD, Honestly significant difference; IQ, Intelligence quotient; ISI, Interstimulus interval; ISV, Intra-subject variability; MNI, Montreal Neurological Institute; MRI, Magnetic resonance imaging; PC, Posterior commissure; PFC, Prefrontal cortex; ROI, Region of interest; SES, Socioeconomic status; SMA, Supplementary motor area; SPSS, Statistical Package for Social Sciences; STD, Standard deviation; SWM, Spatial working memory; TBSS, Tract-based spatial statistics; TD, Typically developing; VLPFC, Ventrolateral prefrontal cortex; WISC, Wechsler Intelligence Scale for Children; WMI, Working Memory Index; WMP, Working memory index (BRIEF parent rating).

## Competing interests

The authors declare that they have no competing interests.

## Authors’ contributions

KLM, RBB, AEC, SL and CAC designed the study. KLM and RBB designed and implemented the fMRI paradigms. CAC with AEC and SL determined the clinical psychology tests to be administered to correlate with the fMRI paradigms. PDG and KLM performed the fMRI studies. KLM and RBB determined the appropriate imaging analysis parameters. RS and JLB determined the appropriate statistical analysis for group comparisons, which were performed by JLB and LWF. JLB, KLM and LWF analyzed the fMRI data. KLM, RBB and JLB interpreted the data. LWF performed the psychological assessments and LWF and CAC analyzed this data and prepared reports on each subject. AEC and SL screened the subjects for MRI and provided clinical expertise. JLB drafted the manuscript with KLM and RBB. All authors read and approved the final manuscript.
